# I Feel Different, but in Every Case I Feel Proud: Distinguishing Self-Pride, Group-Pride, and Vicarious-Pride

**DOI:** 10.3389/fpsyg.2021.735383

**Published:** 2021-11-23

**Authors:** Ilona E. De Hooge, Yvette Van Osch

**Affiliations:** ^1^Department of Marketing and Consumer Behavior, Wageningen University & Research, Wageningen, Netherlands; ^2^Department of Social Psychology, Tilburg University, Tilburg, Netherlands

**Keywords:** pride, self-conscious emotions, vicarious emotions, group-based emotions, collective emotions, self-evaluation, social self, subjective experience

## Abstract

Various lines of research have hinted at the existence of multiple forms of self-conscious emotion pride. Thus far, it is unclear whether forms, such as self-pride, group-pride, or vicarious-pride are characterized by a similar feeling of pride, and what the communal and unique aspects are of their subjective experiences. The current research addressed this issue and examined the communal and unique characteristics of the subjective experiences of self-pride, group-pride, and vicarious-pride. Using recalled experiences, two experiments demonstrated that self-pride, group-pride, and vicarious-pride could be separated on the basis of their subjective experiences. More specifically, Experiment 2 demonstrated how self-pride, group-pride, and vicarious-pride were related to feelings of self-inflation, other-distancing vs. approaching, and other-devaluation vs. valuation. Finally, Experiment 3 showed that not only the responsibility for the achievement but also the number of people who had contributed to the achievement could influence the experience of other-oriented forms of pride. The current findings revealed that self-pride, group-pride, and vicarious-pride were all forms of pride with distinct subjective experiences. These findings provided valuable insights into the emotion of pride and might lead to divergent consequences for sociality, self-consciousness, and behavior.

## Introduction

In daily life, people can feel proud of different things. They may for example feel proud of having achieved a goal (Rinas et al., 2020), of having won a sports game (Van Osch et al., 2016), or of having learned something new (Bellocchi and Ritchie, 2015). Teachers may feel proud of their students having mastered a new subject (Darby, 2008; Myall et al., 2008), parents may feel proud of their children have developed a new skill (Nakamura, 2013; Pasupathi et al., 2020), employees may feel proud of their work team having reached a target (Tyler and Blader, 2001), or fans may feel proud of their sports team has won a championship (Bravo et al., 2020). In all of these cases, one or more people have achieved something positive or valued, and the ensuing positive feelings can be described as feelings of pride (Salice and Montes Sanchez, 2016; Ritzenhofer et al., 2019). Existing literature has provided different labels for such pride experiences. Whereas feeling proud of own achievements (“proud of me”) has been described as self-pride (Delvaux et al., 2016; Septianto et al., 2018), individual pride (Sullivan, 2017), independent pride (Ahuvia et al., 2018), self-referential pride (Ritzenhofer et al., 2019), or authentic pride (Tracy and Robins, 2007a,b), feeling proud of the achievements of the group an individual belongs to, or of a group one associate with (“proud of us”), has been studied as group-pride (Zander and Armstron, 1972; Zander et al., 1972; Delvaux et al., 2016; Thomas et al., 2017), group-based pride (Harth, 2008; Harth et al., 2013; Schori-Eyal et al., 2015; Sullivan, 2017), group-level pride (Williams and Davies, 2017), interdependent pride (Ahuvia et al., 2018), or collective pride (pride felt by a group of people, not an individual; Van Leeuwen et al., 2013; Sullivan, 2018; White and Branscombe, 2019; Bravo et al., 2020). In addition, instances in which people feel proud of the achievements of one or a few other people (“proud of you”) have been examined under the concepts of vicarious pride (Williams and Davies, 2017; Septianto et al., 2018; Ritzenhofer et al., 2019; Yoon and Shanker Krishnan, 2019), parenting pride (Pasupathi et al., 2020), family pride (Sircar et al., 2021), or relational pride (Liu et al., 2014; Sullivan, 2017).

Although these different lines of research have generated valuable insights into the antecedents, experiences, and consequences of each of these forms of pride separately, we are not aware of any studies that have directly compared the subjective experiences of these different forms of pride. Some emotional researchers have speculated whether feeling proud of own achievements would be the same as feeling proud of a group achievement or as feeling proud of the achievement of another person (Salice and Montes Sanchez, 2016; Salmela and Sullivan, 2016; Williams and Davies, 2017), and few studies have compared the consequences of self-pride with vicarious-pride (Decrop and Derbaix, 2010; Septianto et al., 2018; Ritzenhofer et al., 2019). Yet, empirical research on the differences and similarities between the subjective experiences of these various forms of pride remains scarce. Consequently, on a theoretical and empirical level, it is currently unclear whether all of these different forms of pride denote the same or distinct subjective feelings of pride. The current research aims to address this issue by examining whether self-pride, group-pride, and vicarious-pride are characterized by subjective feelings of pride, and by examining what the communal and unique aspects are of their subjective experiences. Moreover, as self-pride, group-pride, and vicarious-pride differ in the degree to which people are responsible for the achievement, we also examine the role of personal responsibility for achievement in feelings of pride.

## Theoretical Background

### Self-Pride

Situational experiences of pride can be described as self-focused feelings that arise after having achieved something good, valued, or virtuous (Smith and Ellsworth, 1985; Haidt, 2003). Pride is a positive, self-conscious emotion as it requires people to be able to reflect upon and evaluate themselves and their actions (James, 1890; Tangney, 1999; Tracy and Robins, 2004). The emotion is generated by appraisals that oneself is responsible for a positive, socially valued outcome (Mascolo and Fischer, 1995; Salice and Montes Sanchez, 2016), and is mostly experienced after a goal-congruent achievement has been ascribed to internal, unstable, and controllable causes, such as effort (Smith and Ellsworth, 1985; Tracy and Robins, 2007a,b).

The function of pride is to provide valuable information about the social status and acceptance of people (Tracy and Robins, 2007b; Bollo et al., 2018). The emotion encourages people to continue the valued behavior (McCullough et al., 2001; Tangney et al., 2007), and to persevere on difficult goals (Williams and DeSteno, 2008; Wilcox et al., 2011). Pride also has a social function, in that it can motivate or strengthen behaviors that are valued within the social group of an individual (Haidt, 2003; Williams and DeSteno, 2009; Dorfman et al., 2014). Other research suggests that self-pride motivates people to seek more status and to engage in positive differentiation from others, for example with high-status purchases (Griskevicius et al., 2010; Bollo et al., 2018; Septianto et al., 2018). Indeed, displays of pride signal high (social) status (Cheng et al., 2010; Shariff et al., 2012), which may generate deference from others (Martens et al., 2012). As self-pride seems to relate to feelings of responsibility for the achievement, to the motivation of valued behavior, and the attainment of status, our research will examine whether these elements also occur for the other forms of pride.

Multiple scholars have made a distinction between more authentic feelings of pride or beta-pride in which people experience pride for a specific behavior or action, from hubris or alpha pride in which people experience pride for themselves in general (also called pridefulness) (Tangney, 1990; Lewis, 1992; Tracy and Robins, 2007a,b; Tracy et al., 2009, 2010). Authentic pride “is typically based on specific accomplishments” (Tracy and Robins, 2007b, p. 507) and involves attributing the achievement to internal, unstable, controllable causes, such as temporary effort (Holbrook et al., 2014; Lange and Crusius, 2015). Hubris or hubristic pride is thought to concern a more individual tendency (Lewis, 1992), or a tendency to attribute achievements to internal, stable, uncontrollable causes, such as personal abilities (Tracy and Robins, 2004; Lange and Crusius, 2015). As the current research mostly focuses on situational experiences of pride, the focus will be on authentic pride. When people experience such pride, they feel pleased, satisfied, and joyous (Frijda et al., 1989; Decrop and Derbaix, 2010; Lange and Crusius, 2015), accomplished and confident (Tracy and Robins, 2007a). Proud people experience feelings of self-worth (Tracy and Robins, 2007b) and self-inflation (Roseman et al., 1996; Van Osch et al., 2018), and it is argued that they also experience an increase in self-esteem (Tracy and Robins, 2007b; Tracy et al., 2009).

### Other-Oriented Forms of Pride

As we have delineated above, more group-focused forms of pride have been described as collective pride, interdependent pride, group-pride, group-based pride, or group-level pride. This group-based form of pride belongs to intergroup or group-based emotions that arise when events occur to a social group with which people identify themselves (Mackie et al., 2008; White and Branscombe, 2019). Intergroup or group-based emotions are thought to differ from individual emotions because group-based emotions depend on the degree of group identification, are shared with other group members, and contribute to the regulation of group attitudes and behavior (Smith et al., 2007; Harth et al., 2008). Similarly, collective pride or national pride concerns group experiences of pride, which is not the concept that we focus upon here (Sullivan, 2014).

Group-pride arises when people categorize themselves to or are affiliated with a group that is responsible for admirable behavior (Liu et al., 2014; Salmela and Sullivan, 2016; Williams and Davies, 2017). The emotion arises especially when the group has put effort into the achievement (Zander et al., 1972) and when the achievement is legitimate (Harth et al., 2008; Williams and Davies, 2017). People are not necessarily *personally* responsible for the admirable behavior or achievement of the group they belong to (White and Branscombe, 2019). As long as people perceive themselves as group members, they can feel entitled to (part of) the achievement and can feel proud of the joint achievement (Salmela and Sullivan, 2016). Group-pride is thought to motivate the pursuit of group-related goals and commitment to the group (Williams and Davies, 2017). Hardly any research has described or studied the subjective experience of this group-based form of pride. Most research on group-pride has presented group-pride as “a feeling of pride for group achievements,” without specifying whether the experience of pride is similar to the experience of self-pride. Few studies have mentioned that experiences of group-pride include feeling associated with or experiencing a sharedness with or intimacy with others (Sullivan, 2013; Delvaux et al., 2016; Bravo et al., 2020). Therefore, the present research will examine whether group-pride can be distinguished from the other forms of pride on feelings of closeness. We will also include tendencies to approach others in general and, as the opposite, tendencies to distance oneself from others in general.

Social or vicarious forms of pride have been previously labeled as parenting or family pride, relational pride, or vicarious pride. Vicarious emotions are emotions that are experienced in response to the actions or situations of one or a few other individuals (Tangney et al., 2007; Wondra and Ellsworth, 2015). The emotions are not based on how people perceive the state or emotions of others, but on how people appraise the situation of the other person (Smith, 1759; Paulus et al., 2013; Wondra and Ellsworth, 2015). Vicarious emotions mostly occur when people feel close to or identify with those others (Lickel et al., 2005; Welten et al., 2012), and when the situation of others is novel, attracts attention, and conveys enough information to appraise the situation (Wondra and Ellsworth, 2015). Vicarious emotions provide information and foster social interactions (Paulus et al., 2013).

Vicarious-pride is felt when another person or a few other people have achieved a positive outcome (Salice and Montes Sanchez, 2016; Ritzenhofer et al., 2019). Vicarious-pride arises especially when the achiever concerns a close or liked other (Williams and Davies, 2017). This form of pride is suggested to motivate goal-pursuit of the observer and social support for the goal-pursuit of the achiever (Williams and Davies, 2017). The emotion would thereby have positive effects on social relationships. This suggestion makes it interesting to study whether vicarious-pride would differ from self-pride or group-pride on feelings of closeness to others or on tendencies to approach others. There are also some suggestions that feeling proud of the achievements of others may be related to feelings of admiration (Williams, 2018; Watkins and Bastian, 2019). We will therefore also examine whether vicarious-pride is related to experiences of admiration. Similar to group-pride, there is no literature that has described or examined the subjective experience of vicarious-pride, or whether the experience of vicarious-pride is similar to experiences of self-pride or group-pride.

We would like to note that it is currently unclear how exactly experiences of pride over achievements made together with a group, which we refer to here as group-pride, relate to experiences of pride over the achievements of one or a few close others, which we refer to as vicarious-pride. The literature overview seems to suggest that these two forms of pride are distinct phenomena (see also Williams and Davies, 2017), and thus we have treated them as such. At the same time, it is uncertain whether this distinction relies on a difference in responsibility for the achievement (partially responsible in the case of group-pride and not responsible in the case of vicarious-pride), or a difference in the number of people contributing to the achievement (a group or larger number of people for group-pride and a single or a few individuals(s) for vicarious-pride), or on something else entirely (e.g., group-pride being an empathic emotion and vicarious-pride being a vicarious emotion, Wondra and Ellsworth, 2015). In our final experiment, we aim to provide some preliminary insights on this issue.

### Comparisons Between Different Forms of Pride

Only a few empirical studies have compared the different forms of pride. These studies have mostly focused on idiosyncratic outcomes of self-pride and vicarious-pride. For example, one study showed that self-pride activates a more competitive mindset (a stronger focus on a status motive) and a lower collaborative mindset (a weaker focus on an affiliation motive) compared to vicarious-pride (Septianto et al., 2018). Self-pride has also been found to relate more to ascribed agency and perceived autocratic leadership, and to relate less to perceived communality and perceived democratic leadership compared to vicarious-pride (Ritzenhofer et al., 2019). In their research on sports consumption, Decrop and Derbaix (2010) found that feeling proud of being a fan (self-pride) is related to building an own identity or boosting own self-confidence, whereas feeling proud of a sports’ team that an individual is a fan of (labeled vicarious-pride) is related to the creation of a collective self. On a theoretical level, both Salice and Montes Sanchez (2016) and Williams and Davies (2017) have argued that self-pride and other-oriented (hetero-induced) forms of pride generate similar feelings of pride. These other-oriented forms of pride would also generate similar tendencies to show off (Salice and Montes Sanchez, 2016), and would have similar stimulating effects on goal-pursuit, although different mechanisms may underlie these effects (Williams and Davies, 2017). Overall, based on these empirical studies and theoretical accounts, we would expect both differences and commonalities in the subjective experiences of these different forms of pride.

One essential question, rarely empirically addressed before, is whether it is necessary to feel responsible for the achievement or positive outcome in order to experience feelings of pride (Williams and Davies, 2017). Being responsible for a positive outcome is at the core of most definitions of self-pride (e.g., Smith and Ellsworth, 1985; Tangney and Fischer, 1995; Tracy and Robins, 2007a,b). On the contrary, some scholars have suggested that different forms of pride are possible, as long as the person perceives to be at least partially responsible for the achievement (Walsh, 1970). Other scholars have suggested that people may not feel personally responsible for the achievement but can still experience feelings of pride when they identify themselves as a member of the group to which those who act admirably also belong (Walsh, 1970; Decrop and Derbaix, 2010; Salice and Montes Sanchez, 2016), or when they experience closeness with or relatedness to the achiever (Salice and Montes Sanchez, 2016; Ritzenhofer et al., 2019). We expect perceived responsibility to be a differentiating element between self-pride and other-oriented forms of pride, such that perceived responsibility plays a stronger role in experiences of self-pride than in experiences of group-pride and vicarious-pride.

In sum, multiple lines of research have demonstrated the existence and consequences of self-pride, group-pride, and vicarious-pride, but hardly any research has empirically compared the experiential contents of these various forms of pride. Also, it is currently unclear what the subjective experiences of group-pride and vicarious-pride entail, and whether these experiences are similar to experiences of self-pride. Finally, it is unclear whether feeling responsible for an achievement or identifying with an achiever is necessary to experience feelings of pride.

The current research addresses these issues and examines the subjective experiences of people and feelings of responsibility for the achievement following in-vivo experiences of self-pride, group-pride, and vicarious-pride. To manipulate the three forms of pride, we followed the definitions of Williams and Davies (2017) of the three concepts of being proud of oneself for being successful (self-pride), being proud of oneself and their group for being successful (group-pride), and being proud of another person because (s)he was successful (vicarious-pride). All three experiments examined feelings of pride and feelings of being responsible for the achievement following these manipulations. Following the suggestions that all forms of pride would motivate people (Williams and Davies, 2017), that especially self-pride would relate to increased status (Griskevicius et al., 2010; Bollo et al., 2018; Septianto et al., 2018), and that especially other-oriented forms of pride would relate to increased closeness to others (Delvaux et al., 2016; Williams and Davies, 2017; Bravo et al., 2020) and increased admiration for others (Williams, 2018; Watkins and Bastian, 2019), Experiment 1 examined the motivation of people, their felt status, change in closeness to others, and admiration following the three forms of pride. Experiments 2 and 3 more closely examined the interplay of the self and others and the tendencies to inflate or deflate oneself or others following the different forms of pride and studied whether the experiences of people when it comes to self- and other-inflation, of distancing and approaching and of other-appreciation and -devaluation were affected differently by the different forms of pride. In addition, Experiment 3 more closely studied the role of responsibility for the achievement and distinguished the aspect of responsibility from the number of achievers in the other-oriented forms of pride. Experiment 1 was of a more exploratory nature, whereas Experiments 2 and 3 were preregistered on aspredicted.org.

## Experiment 1

The goal of Experiment 1 was to examine whether the three forms of pride could be distinguished in their subjective experiences. As there is at present no research providing a clear overview of the differences in subjective experiences between the three forms of pride, the current experiment was of an exploratory nature. We manipulated self-pride, group-pride, and vicarious-pride following the idea that self-pride focuses on feeling proud of own achievements, that group-pride focuses on feeling proud of shared achievement, and that vicarious-pride focuses on feeling proud of the achievement of another person (Williams and Davies, 2017). We then measured the experienced pride of people (in two different ways) (Van Osch et al., 2018, 2019), authentic pride (Tracy and Robins, 2007b), responsibility for the achievement, motivation (Williams and Davies, 2017), experienced status (Shariff et al., 2012), admiration (Onu et al., 2016; Williams, 2018), and closeness to others (Delvaux et al., 2016; Bravo et al., 2020).

### Materials and Methods

#### Participants and Design

Our aim was to collect as much data as possible for this experiment in 3 weeks. Among the 290 participants starting the research, 58 participants terminated the research before answering the dependent variables, and one participant did not answer the manipulation. The final sample consisted of 231 first-year psychology students (66 men, 165 women; *M*_age_ = 20.23, *SD*_age_ = 2.14; 179 Dutch students, 52 International students). The participants were recruited online via the university lab portal and completed the study for partial course requirements. They were randomly assigned to one of the four conditions (Self-pride, Group-pride, Vicarious-pride, or Control condition) of a between-subjects design.

#### Procedure

Dutch students completed the study in Dutch and International students completed the study in English.^1^ Before the data collection took place, two undergraduate students translated and back-translated all of the study materials. Inconsistencies in translations were resolved through discussion between the students and the authors. After providing informed consent, the participants first fulfilled an autobiographical recall manipulation as the emotion manipulation method. The participants continued with providing their answers to the dependent measures.^2^ At the end of the study, the participants were debriefed and given the opportunity to provide comments.

#### Manipulation

We used an autobiographical recall procedure to manipulate experiences of pride (De Hooge et al., 2007; Van Osch et al., 2018). An autobiographical recall procedure involves participants remembering a personal, emotional experience and describing details of the experience to reactivate the experienced emotions (Prkachin et al., 1999; Siedlecka and Denson, 2019). In the present experiment, the participants were asked to recall and describe either (1) an experience in which they were proud of themselves for being successful (Self-pride condition), (2) an experience in which they were proud of themselves and their group for being together successful at something (Group-pride condition), (3) an experience in which they were proud of someone else or a group of others because they were successful at something (Vicarious-pride condition), or (4) a regular weekday on which they saw at least one friend or family member (Control condition).^3^ In all conditions, the participants were asked to recall as much detail as possible about this experience and to write a short story to explain this experience to someone who had not been present. Participants in the self-pride condition most often recalled situations in which they achieved academic success or overcame hardship. Participants in the group-pride condition most often described academic or athletic team achievements, and participants in the vicarious-pride condition almost always described instances in which a close other had achieved academic or athletic success or overcame personal hardship.

#### Measures

All the variables were measured on 7-point Likert scales ranging from 1 (Not at all) to 7 (Very much). To measure experienced pride and perceived responsibility, the participants reported their levels of *experienced pride* (“In this situation, I felt pride/joy/satisfaction,” α = 0.83; Van Osch et al., 2018, 2019),^4^ *authentic pride* (e.g., “accomplished”; 7 items; α = 0.94; Tracy and Robins, 2007b), and perceived level of *responsibility* (“I felt responsible for the described achievement”).

The participants then reported the extent to which they felt *motivated* (“The situation motivated/stimulated/inspired me”; 3 items; α = 0.89), the extent to which they perceived that they had attained *status* in the eyes of other(s) in the described situation (e.g., “I thought others thought of me as important”; 6 items; α = 0.88; Dijkstra et al., 2010; Shariff et al., 2012), and the extent to which they felt *admiration* (e.g., “In this situation I felt admiration for others”; 3 items; α = 0.91; adapted from Onu et al., 2016). In the group-pride and vicarious-pride conditions, we additionally asked the participants to report how close they felt to the other(s) both before and after the experience had occurred (“How close was your relationship before/after the described situation?”). We calculated a difference score from the closeness before and after the situation had occurred. Positive scores on this *closeness* measure reflected an increase in closeness.

Finally, we asked the participants to indicate what kind of relationship they had with the other(s) (nuclear family member, extended family member, friend, acquaintance, fellow countrymen, not personally, or other). In the group-pride condition, 78% of the participants indicated that the experience concerned friends. In the vicarious-pride condition, 41% of the participants reported an experience concerning a nuclear family member and 48% reported an experience concerning a friend.

### Results

#### Pride and Responsibility

For all measures, we conducted One-way ANOVAs with Condition as the independent variable and the measure as the dependent variable, and with Bonferroni corrections to correct for potential Type-I errors (see Table 1). Analyses of *experienced pride* (both the three-item measure and the single pride item) and *authentic pride* revealed that the participants reported more pride in all pride conditions than in the Control condition. This suggests that the manipulation had aroused feelings of pride. The participants reported feeling more authentic pride in the Self-pride and Group-pride conditions than in the Vicarious-pride and Control conditions. The participants reported feeling most *responsible* in the Self-pride condition, followed by the Group-pride and the Control condition. The participants reported the least responsibility for the achievement in the Vicarious-pride condition.

**TABLE 1:** Means, standard deviations, and statistics of all dependent variables in Experiment 1.

	**Experimental condition**					
	**Self-pride (*n* = 54)**	**Group-pride (*n* = 59)**	**Vicarious-pride (*n* = 61)**	**Control (*n* = 57)**		
**Pride and responsibility**	***M* (*SD*)**	***M* (*SD*)**	***M* (*SD*)**	***M* (*SD*)**	***F*(1, 223)**	**η ^2^**

Experienced pride (3 items)	6.51 (0.77)_a_	6.18 (1.01)_a_	6.13 (0.93)_a_	5.03 (1.34)_b_	11.62*	0.14
Experienced pride (1 item)	6.46 (0.88)_a_	6.19 (0.90)_a_	6.48 (0.74)_a_	4.19 (1.57)_b_	40.82*	0.35
Authentic pride	6.09 (0.84)_a_	5.77 (1.06)_a_	4.58 (1.25)_b_	4.58 (1.13)_b_	20.83*	0.22
Responsibility	6.10 (1.35)_a_	5.29 (1.46)_b_	3.47 (2.00)_c_	4.53 (1.41)_b_	18.05*	0.21

**Dependent measures**	***M* (*SD*)**	***M* (*SD*)**	***M* (*SD*)**	***M* (*SD*)**		

Motivation	5.89 (1.15)_a_	5.81 (1.00)_a_	5.69 (0.96)_a_	4.36 (1.43)_b_	10.87*	0.13
Status	3.31 (1.23)_ab_	3.81 (1.33)_b_	2.99 (1.38)_a_	2.96 (1.12)_a_	5.59*	0.07
Admiration	3.38 (1.31)_a_	4.67 (1.34)_b_	5.19 (1.35)_b_	3.47 (1.46)_a_	11.28*	0.13
Difference closeness		0.95 (1.24)_a_	0.36 (0.71)_b_		4.34^†^	0.04

Means with different subscripts differed significantly in pairwise comparisons (Bonferroni corrected α = 0.05/7 = 0.007), *p = 0.001, ^†^*p* < 0.05.

#### Dependent Variables

As can be seen in Table 1, in all pride conditions the participants reported being more *motivated* by the experience than the participants in the Control condition. The participants in the Group-pride condition reported significantly higher perceptions of having attained *status* than those in the Vicarious-pride and Control conditions. The Self-pride condition did not differ from any other condition on perceptions of having attained status. The participants in the Group-pride and the Vicarious-pride condition reported feeling more *admiration* for others than those in the Self-pride and Control conditions. Finally, the participants in the Group-pride conditions reported a stronger increase in *closeness* with the other(s) after the experience than those in the Vicarious-pride condition.

### Discussion

The findings of Experiment 1 reveal that people may experience similar feelings of pride after a self-pride, group-pride, or vicarious-pride experience, even though they differ in the degree to which they feel responsible for the achievement. Whereas, people feel strongest personally responsible for having individually achieved success, they feel least responsible for a success achieved by another person. Surprisingly, our results also showed stronger feelings of authentic pride for self-pride and group-pride experiences than for vicarious-pride experiences. This may be the case because the measure of authentic pride, which was originally developed to measure feelings of self-pride (Tracy and Robins, 2007b), contains multiple items related to feeling agentic and responsible for the achievement. Indeed, in our study the measures of responsibility and authentic pride were strongly correlated, *r*(209) = 0.55, *p* < 0.001. One may thus wonder whether this measure of authentic pride is suitable to capture feelings of pride for other-oriented achievements.

The findings also reveal that self-pride, group-pride, and vicarious-pride may not differ in the degree to which they motivate people, but they may differ in generating perceptions of having attained status, in feeling admiration for others, and in feeling close to others. Whereas, group-pride seemed to mostly increase own perceptions of having attained status, both group-pride and vicarious-pride experiences seemed to elicit more feelings of admiration for others. Also, group-pride seems to bring people closer together than experiences of vicarious-pride.

This first exploratory study shows that pride experiences may affect both how people feel and think about themselves, and how they feel and think about other people. Previous research has demonstrated that self-pride mainly affects how people feel about themselves and hardly how people feel about others (Van Osch et al., 2018). Experiment 2 aimed to replicate the findings of Experiment 1 and to examine how self-pride, group-pride, and vicarious-pride affect the feelings of people toward themselves and feelings toward others.

## Experiment 2

Experiment 2^5^ employed an identical design to Experiment 1 but focused on dependent variables related to feelings toward oneself and toward other(s) during the pride experience. We aimed to replicate the finding from Experiment 1 that people during all forms of pride experiences had stronger pride feelings than people during a regular weekday (H1), and that people perceived themselves to be less responsible for the achievement during a vicarious-pride experience than during a self-pride or group-pride experience (H2). Moreover, previous literature has suggested that authentic feelings of self-pride elicit bodily and psychological experiences of self-inflation (Roseman et al., 1996; Van Osch et al., 2018). The experience of an inflated self relates to feeling personally responsible for the achievement (Van Osch et al., 2018). Since people tend to feel at least some responsibility for achievement when experiencing group-pride (Salmela and Sullivan, 2016; Williams and Davies, 2017) and may feel even less responsible for achievement when experiencing vicarious-pride (H2), it was expected that self-pride would be likely to elicit more self-inflation than group-pride, which in turn would elicit more self-inflation than vicarious-pride (H3). We also explored whether vicarious-pride and group-pride would generate the opposite tendency, that is a tendency to inflate others or, as demonstrated in Experiment 1, to admire others. Self-pride has also been related to differentiating oneself from others (Griskevicius et al., 2010; Bollo et al., 2018; Septianto et al., 2018), and group-pride has been related to feeling associated with others (Delvaux et al., 2016; Bravo et al., 2020). We, therefore, expected people experiencing group-pride and vicarious-pride to perceive themselves as closer to others and to be more likely to approach others than people experiencing self-pride (H4). Finally, we measured distancing from others and other-devaluation (Van Osch et al., 2018), and benign and malicious envy (Van de Ven, 2017) to explore whether any form of pride would generate such negative feelings toward others.

### Method

#### Participants and Design

A g-power analysis based on a small to medium effect size (η^2^ = 0.10; 0.80 power; alpha = 0.05) showed that 104 participants were needed. However, because this study ran together with another study that needed 210 participants, we collected 212 participants. Six participants terminated the study before the end of the study, nine participants failed the attention check, and two participants indicated afterward that they had misunderstood the instructions. The final sample consisted of 195 first-year psychology students (171 women, 1 unknown; *M*_age_ = 19.37, *SD*_age_ = 2.57) that were recruited online via the university lab portal. They completed the study for partial course requirements. The participants were randomly assigned to one of the four conditions (Self-pride vs. Group-pride vs. Vicarious-pride vs. Control) of a between-subjects design.

#### The Procedure, Manipulation, and Measures

After having provided informed consent, the participants fulfilled the manipulation procedure of Experiment 1. The types of experiences described within the different conditions were very similar to what we observed in Experiment 1. The participants then continued with providing their answers to the dependent measures (all measured on 7-points Likert scales ranging from 1 = Not at all to 7 = Very much). The participants reported their levels of perceived *self-inflation* (e.g., “I felt important”; 7 items; α = 0.88), *distancing* from others (e.g., “I wanted to separate myself from others”; 4 items; α = 0.83), and *other-devaluation* (e.g., “I thought others were less than me”; 5 items; α = 0.83) (all derived from Van Osch et al., 2018). To develop analogous measures that would tap into the phenomenological experiences of group-pride and vicarious-pride, we rephrased the items mentioned above to reflect on the other(s) who had achieved something. This resulted in scales tapping into perceived *other-inflation* [e.g., “The other(s) seemed large”; 7 items; α = 0.92], *approaching others* [e.g., “I wanted to be together with the other(s)”; 4 items; α = 0.84], and *other-appreciation* (e.g., “I thought the other was valuable”; 5 items; α = 0.91).

Experienced pride was measured with the same measure as in Experiment 1 (α = 0.83 for the three-item measure). We created a parallel measurement for perceived pride of the other(s) (“In this situation, the other(s) felt pride/joy/satisfaction”; α = 0.88). Perceived responsibility was measured with the single item from Experiment 1. For exploratory purposes, we included single-item measures of admiration (“I admired the other”), benign envy, and malicious envy (Van de Ven, 2017).^6^

### Results

#### Pride and Responsibility

For all measures, we conducted One-way ANOVAs with Condition as the independent variable and the measure as the dependent variable with Bonferroni corrections (see Table 2). Confirming H1, the ANOVAs on Experienced pride (single-item measure and three-item measure) both showed that the participants reported more pride feelings in all pride conditions compared to the Control condition. The participants in the Group-pride and Vicarious-pride conditions also perceived more pride in the other(s) than in the Self-pride and Control conditions. All conditions differed from each other in terms of perceived responsibility for the achievement. The participants reported feeling most responsible in the Self-pride condition, followed by the Group-pride condition, and then by the Control condition. The participants felt least responsible for the achievement in the Vicarious-pride condition, confirming H2.

**TABLE 2:** Means, standard deviations, and statistics of all dependent variables in Experiment 2.

	**Experimental condition**					
	**Self-pride (*n* = 46)**	**Group-pride (*n* = 51)**	**Vicarious-pride (*n* = 49)**	**Control (*n* = 49)**		
**Pride and responsibility**	***M* (*SD*)**	***M* (*SD*)**	***M* (*SD*)**	***M* (*SD*)**	***F*(3, 191)**	**η ^2^**

Experienced pride (3 items)	6.39 (0.72)_a_	6.27 (1.11)_a_	6.16 (0.86)_a_	5.22 (0.94)_b_	16.37*	0.21
Experienced pride (1 item)	6.46 (0.75)_a_	6.31 (1.19)_a_	6.47 (0.65)_a_	4.43 (1.17)_b_	50.53*	0.44
Pride of other	5.62 (1.38)_a_	6.34 (0.93)_b_	6.20 (1.17)_b_	5.29 (0.70)_a_	10.39*	0.14
Responsibility	6.65 (0.67)_a_	5.78 (1.12)_b_	2.41 (1.71)_c_	4.78 (1.31)_d_	101.33*	0.61

**Dependent variables**	***M* (*SD*)**	***M* (*SD*)**	***M* (*SD*)**	***M* (*SD*)**	***F*(3, 191)**	**η ^2^**

Self-inflation	5.07 (0.96)_a_	4.41 (1.12)_b_	2.75 (1.18)_c_	3.50 (0.87)_d_	45.86*	0.42
Other-inflation	3.30 (1.23)_a_	4.71 (1.07)_b_	5.94 (0.71)_c_	3.87 (1.16)_d_	56.52*	0.47
Distancing	3.10 (1.34)_a_	2.21 (1.10)_b_	1.55 (0.75)_c_	2.14 (1.03)_b_	16.82*	0.21
Approach	3.49 (4.55)_a_	4.60 (1.29)_b_	5.01 (1.52)_b_	4.36 (1.04)_b_	10.45*	0.14
Devaluation	1.30 (0.55)_a_	1.23 (0.67)_a_	1.12 (0.34)_a_	1.20 (0.46)_a_	0.91	0.01
Other-appreciation	3.72 (1.50)_a_	5.10 (1.40)_b_	6.09 (0.67)_c_	4.64 (1.38)_b_	28.49*	0.31
Admiration	3.46 (2.05)_a_	5.31 (1.44)_b_	6.45 (1.84)_c_	4.67 (1.60)_b_	32.57*	0.34
Benign envy	2.20 (1.75)_ab_	1.92 (1.45)_a_	2.90 (2.01)_b_	2.47 (1.43)_ab_	3.07^†^	0.05
Malicious envy	1.59 (1.19)_a_	1.35 (0.93)_a_	1.43 (0.68)_a_	1.63 (0.95)_a_	0.95	0.02

Means with different subscripts differed significantly in pairwise comparisons (Bonferroni corrected α = 0.05/13 = 0.004), *p < 0.001, ^†^*p* < 0.05.

#### Dependent Variables

As can be seen in Table 2, we observed significant differences between the four conditions on all the dependent variables, except for devaluation and malicious envy. We will first discuss the differences between self-pride and the other forms of pride and then turn to differences between group-pride and vicarious-pride.

##### Comparing Self-Pride With Group-Pride and Vicarious-Pride

As hypothesized in H3, the participants reported more self-inflation in the Self-pride condition than in Group-pride, Vicarious-pride, and Control conditions. The participants in the Self-pride condition also reported more feelings of distancing from others and fewer feelings of approaching others than the participants in the three other conditions. This finding supported H4. Finally, the participants in the Self-pride condition reported less other-inflation, other-appreciation, and admiration than the participants in the other three conditions.

##### Comparing Group-Pride With Vicarious-Pride

The participants in the Group-pride condition reported more self-inflation and distancing from others compared to the participants in the Vicarious-pride condition. They also reported less other-inflation, other-appreciation, admiration, and benign envy compared to the participants in the Vicarious-pride condition. The two other-oriented forms of pride did not differ in approaching others.

### Discussion

The findings of Experiment 2 reveal that other-oriented forms of pride can be distinguished from self-pride on feelings of responsibility, feelings toward oneself, and feelings toward other people. Replicating the findings of Experiment 1, when people experience self-pride, they feel more responsible for the achievement, and experience more positive feelings toward themselves (e.g., more self-inflation) compared to the other-oriented forms of pride. In addition, Experiment 2 reveals that self-pride is associated with less strong positive feelings toward others (e.g., more distancing from others, lesser other-inflation, other-appreciation, and admiration) compared to other-oriented forms of pride. Replicating the findings of Experiment 1, the findings of Experiment 2 confirm that group-pride and vicarious-pride can be distinguished from each other. Similar to the results of Experiment 1, group-pride leads to stronger feelings of responsibility for the achievement and more positive feelings toward oneself (e.g., more self-inflation) compared to vicarious-pride. On the contrary, Experiment 2 reveals that vicarious-pride leads to more positive feelings toward others (e.g., more other-inflation, other-appreciation, admiration, and benign envy) than group-pride. No such difference appeared when studying the effects of group-pride and vicarious-pride on admiration in Experiment 1. Experiment 3 aimed to shed some more light on the role of responsibility for the achievement and of the number of achievers in the other-oriented forms of pride.

## Experiment 3

In the previous experiments, the definitions of Williams and Davies (2017) of the three concepts of being proud of oneself for being successful (self-pride), being proud of oneself and their group for being successful (group-pride), and being proud of another person because they were successful (vicarious-pride) were used to generate experiences of pride. These definitions converge with the definitions of group-pride and vicarious-pride that are most often used in the literature (e.g., Harth, 2008; Schori-Eyal et al., 2015; Salice and Montes Sanchez, 2016; Salmela and Sullivan, 2016; Septianto et al., 2018). However, these definitions of group-pride and vicarious-pride vary on both the level of responsibility for the achievement (whether the person experiencing pride is partially responsible or not responsible for the achievement) and on the number of people contributing to the achievement (whether a group of other people or one other person has contributed to the achievement). Experiment 3 studied whether the level of responsibility or the number of achievers were the driving force behind the observed differences between group-pride and vicarious-pride in Experiments 1 and 2. In this preregistered experiment,^7^ both of these factors were manipulated and their influences on the variables that had differentiated group-pride and vicarious-pride in Experiments 1 and 2 were examined. We thereby focused on experienced pride and level of responsibility (from Experiment 1), self-inflation, other-inflation, approaching and distancing from others, and other-appreciation (from Experiment 2), and on having attained the status and changes in closeness to others (from Experiment 1). Moreover, to extend the generalizability of the findings, Experiment 3 used a different sample compared to Experiments 1 and 2, focusing on working adults rather than first-year psychology students.

### Method

#### Participants and Design

A g-power analysis based on a small effect size (*f* = 0.20; power = 0.80; α = 0.05) showed that 235 participants were needed. Taking into account the pre-registered exclusion criteria, we collected 335 Prolific participants. Prolific is a data collection platform that enables the data collection from adults living in the United Kingdom. Fifty-six participants did not complete the study, two participants failed the attention check, and 83 participants failed the manipulation check (see below). The final sample consisted of 194 participants (131 women, 1 unknown; *M*_age_ = 32.75, *SD*_age_ = 11.66). The participants were randomly assigned to one of the four conditions of a 2 (Responsibility: not vs. partial) × 2 (Achiever: individual vs. group) between-subjects design.

#### Procedure and Manipulation

After having given informed consent, the participants fulfilled the autobiographical recall procedure. In all of the conditions, the participants were asked to recall and describe an experience in which they felt very proud. In the Individual conditions, the experience focused on achievement of somebody else for which the participant was not responsible (Individual Not-responsible condition), or on achievement of somebody else and the participant together for which they were both responsible (resembling a team achievement; Individual Partial-responsible condition). In the Group conditions, the experience focused on achievement of a group of other people to which the participant did not belong and for which the participant was not responsible (Group Not-responsible condition), or on achievement of a group of other people and the participant for which they were all responsible (Group Partial-responsible condition). In the Individual Not-responsible condition, the participants described experiences in which a close other had achieved academic or athletic success or overcame personal hardships, much like the experiences described in the vicarious-pride conditions in Experiments 1 and 2. In the Individual Partial-responsible condition, the participants described experiences relating to themselves and their partner, a relative, a friend, or colleague achieving something together. In the Group Not-responsible condition, the participants referred to groups of others (both close others, such as family members or friends/colleagues, as well as distant others, such as national sports teams, refugees, or fellow countrymen) achieving something athletically or morally (for example, dealing with national crises, contributing to good causes). Finally, in the Group Partial-responsible condition, the accounts referred to academic or professional group efforts, similar to the experiences described in the group-pride conditions in Experiments 1 and 2. After writing down their recalled experience the participants continued with the dependent measures.

#### Measures

The participants continued with providing their answers to the dependent measures (all on 7-points Likert scales ranging from 1, Not at all, to 7, Very much, all from Experiments 1 and 2). They provided their level of *experienced pride* (α = 0.92) and perceived *responsibility* (1 item) (both from Experiments 1 and 2), and answered the measures of *self-inflation* (α = 0.94), *other-inflation* (α = 0.87), *distancing* (α = 0.83), *other-appreciation* (α = 0.89; all from Experiment 2), perceived *status* (α = 0.94; Experiment 1), and change in *closeness* due to the experience (*r* = 0.78; Experiment 1).

A manipulation check was included to ascertain that the participants had recalled an event in which they attributed responsibility to the correct achiever. The participants were asked to indicate whether one other individual, they and one other individual, multiple other people (excluding themselves), or they and multiple other people had achieved something successful. Most exclusions based on this manipulation check occurred in the Not-responsible Group condition. In this condition, many participants chose the “me and multiple other people” option instead of “multiple other people (without me)” (*n* = 30). The contents of their recalled experiences confirmed that these participants had remembered an experience in which they were partially responsible for the achievement, or took some responsibility for the achievement. These participants were excluded from the analyses.

### Results

#### Pride and Responsibility

For all measures we conducted Two-way ANOVAs with Responsibility and Achiever as the independent variables and the measure as the dependent variable (see Table 3). Again, we corrected for multiple tests and used α = 0.05/8 = 0.006. The ANOVA on *experienced pride* revealed only a main effect of Achiever [*F*(1, 190) = 8.44, *p* = 0.004, partial η^2^ = 0.04; Responsibility and interaction *F*s < 2.43, *p*s > 0.120]. The participants were prouder of a group achievement (*M* = 6.51, *SD* = 0.63) than of an individual achievement (*M* = 6.25, *SD* = 0.76). The ANOVA on *responsibility* showed that the participants in the Not-responsible conditions felt less responsible for the achievement (*M* = 2.69, *SD* = 1.68) than the participants in the Partial-responsible conditions [*M* = 6.01, *SD* = 1.18; *F*(1, 190) = 257.11, *p* < 0.001, partial η^2^ = 0.57]. There was no significant effect of Achiever [*F*(1, 190) = 3.07, *p* = 0.081], nor an interaction effect [*F*(1, 190) = 0.52, *p* = 0.470].

**TABLE 3:** Means and standard deviations of all dependent variables in Experiment 3.

	**Experimental condition**			
	**Not-responsible**		**Partial-responsible**	
	**Individual (*n* = 32)**	**Group (*n* = 61)**	**Individual (*n* = 50)**	**Group (*n* = 51)**
**Pride and responsibility**	***M* (*SD*)**	***M* (*SD*)**	***M* (*SD*)**	***M* (*SD*)**

Experienced pride (3 items)	6.07 (0.89)	6.50 (0.65)	6.37 (0.66)	6.53 (0.62)
Experienced pride (1 item)	6.07 (0.89)	6.50 (0.65)	6.37 (0.66)	6.53 (0.62)
Responsibility	2.34 (1.62)	2.87 (1.70)	5.90 (1.15)	6.12 (1.21)

**Dependent variables**	***M* (*SD*)**	***M* (*SD*)**	***M* (*SD*)**	***M* (*SD*)**

Self-inflation	1.74 (0.71)	2.21 (1.37)	4.35 (1.26)	4.32 (1.34)
Other-inflation	5.58 (0.87)	5.39 (1.32)	4.96 (1.28)	4.72 (1.09)
Distancing	1.68 (0.87)	1.57 (1.03)	1.90 (1.23)	1.75 (0.84)
Other-appreciation	6.06 (1.00)	6.11 (1.25)	5.95 (0.99)	5.96 (1.11)
Admiration	6.16 (0.92)	6.21 (1.50)	5.86 (1.11)	5.94 (1.24)
Status	1.94 (1.44)	2.26 (1.25)	4.11 (1.42)	3.62 (1.47)
Difference closeness	0.88 (1.54)	0.16 (0.55)	1.04 (1.33)	0.62 (1.19)

#### Dependent Variables

A main effect of Responsibility was observed for *self-inflation* [*F*(1, 190) = 163.82, *p* < 0.001, partial η^2^ = 0.46]. The participants reported more self-inflation when they were partially responsible (*M* = 4.34, *SD* = 1.29) than when they were not responsible for the achievement (*M* = 2.05, *SD* = 1.21). There was no main effect for Achiever nor an interaction effect on self-inflation (*F*s < 1.84, *p*s > 0.176). Responsibility also had an effect on *other-inflation* [*F*(1, 190) = 13.47, *p* < 0.001, partial η^2^ = 0.07]. The participants reported more other-inflation when they were not responsible (*M* = 5.45, *SD* = 1.18) than when they were partially responsible for the achievement (*M* = 4.84, *SD* = 1.19). Again, there was no main effect for Achiever nor an interaction effect (*F*s < 1.44, *p*s > 0.232).

A main effect of Responsibility was also observed for perceived *status* [*F*(1, 190) = 74.05, *p* < 0.001, partial η^2^ = 0.28]. The participants reported a higher perceived status when they were partially responsible (*M* = 3.87, *SD* = 1.46) than when they were not responsible for the achievement (*M* = 2.15, *SD* = 1.32). The main effect of Achiever on perceived status was not significant [*F*(1, 190) = 0.17, *p* = 0.682], nor was the interaction effect [*F*(1, 190) = 3.89, *p* = 0.05].

Achiever did have a main effect on change in *closeness* [*F*(1, 190) = 11.15, *p* = 0.001, partial η^2^ = 0.06]. The participants reported a larger change in closeness due to the experience when the achievement concerned an individual (*M* = 0.98, *SD* = 1.41) rather than a group of others (*M* = 0.37, *SD* = 0.92). More specifically, the participants felt closer after having felt proud of an individual who had achieved something than after having felt proud of a group who had achieved something. There was no effect of Responsibility [*F*(1, 190) = 3.36, *p* = 0.068], nor an interaction effect [*F*(1, 190) = 0.74, *p* = 0.390]. Finally, none of the main or interaction effects were significant for the dependent variables *distancing* (*F*s < 1.78, *p*s > 0.184), *other-appreciation* (*F*s < 0.65, *p*s > 0.42), or *admiration* (*F*s < 2.35, *p*s > 0.127).

### Discussion

The findings reveal that the level of responsibility for an achievement matters in the subjective experiences of self-inflation and perceived status. When people feel partially responsible for achievement, they tend to experience increased feelings of self-inflation and higher perceived status than when people are not responsible for the achievement. The number of people have contributed to achievement also plays a role. When people have achieved something together with another individual, or when solely another individual has achieved something, people feel relatively less pride, but at the same time feel closer to the individual compared to a group having achieved something. It thus seems that both the level of responsibility for an achievement (being partially responsible or not responsible) and the number of achievers (one individual or a group of people) independently contribute to the experience of other-oriented forms of pride.

## General Discussion

It appears that pride can be a multifaceted emotion with multiple subjective experiences. When one feels proud of own accomplishments, the emotion increases positive feelings toward oneself and decreases positive feelings toward others. When one feels proud of shared accomplishments, the emotion increases feelings of having attained the status and positive feelings toward oneself and may increase closeness to others and admiration for others. These feelings especially occur when one feels at least partially responsible for the accomplishment. Finally, when one feels proud of the accomplishments of another person or other people, the emotion especially generates positive feelings about others. In addition, this feeling may increase closeness to the achiever when the achiever is a single individual. Together, these findings provide some new insights into the subjective experiences of self-pride, group-pride, and vicarious-pride.

### Theoretical Implications

The present findings show that there are differences in the experiential contents of self-pride and other-oriented forms of pride. While all forms of pride generate similar feelings of pride and motivate people to act, particularly self-pride and vicarious-pride seem to have opposite consequences for oneself and others. Self-pride appears to mostly *increase* self-inflation and to *decrease* other-inflation, other-appreciation, and admiration for others. It also increases the perceived distance between oneself and others. On the contrary, vicarious-pride mostly *decreases* self-inflation and *increases* the pride of others, other-inflation, other-appreciation, and admiration for others. Group-pride is in between these two forms of pride, generating on the one hand self-inflation and perceived status, and on the other hand pride of and admiration for others. These findings may suggest that pride generates a stronger focus on and positive feelings toward the achiever(s). Whereas in self-pride this concerns oneself, in vicarious-pride this concerns another person or other people, and in group-pride this concerns oneself and other people. Consequently, self-pride, group-pride, and vicarious-pride may also differ in the behaviors toward oneself and toward others that may follow the experience. An interesting future direction would be to investigate whether and how the different forms of pride would lead to different behavioral outcomes relating to prosociality. For example, one may expect group-pride experiences to mostly result in enhanced reciprocity or prosociality toward groups, and vicarious-pride to mostly result in enhanced reciprocity or prosociality toward specific individuals.

Although there are many sources that argue the emotion pride to be a sin or sinful (for example, the Bible labeling pride one of the Seven Sins, or Dante (1308–1321/1937) even labeling it the Deadliest of the Seven Deadly Sins), the present research shows that such a negative perspective does not necessarily have to apply to all forms of pride. Instead, our findings reveal that especially vicarious-pride can be something positive. Vicarious-pride does not need to include any negative connotation related to narcissism or selfishness, because it focuses on the performances of others. Moreover, vicarious-pride can be seen as a prosocial emotion. It positively relates to outcomes that positively reflect on others, such as other-inflation, admiration for others, and appreciation of others, and is thereby likely to enhance social relationships. There are some indications that in Eastern cultures, expressions of pride concerning personal accomplishments are regarded as inappropriate or negative (Van Osch et al., 2013), and that expressions of pride concerning accomplishments of others are regarded as positive (Stipek, 1998). This suggests that vicarious-pride may be perceived as more prosocial and acceptable than self-pride.

The current research sheds some light on the role of responsibility for the achievement and the role of the number of people contributing to the achievement in the experience of pride. Previous research has described other-oriented forms of pride in various ways, thereby remaining unclear about whether a person should experience at least partial responsibility for achievement to experience the pride, and about whether the achievement should depend on one or multiple other people to experience group-pride or vicarious-pride. The findings of Experiment 3 reveal that people do not need to perceive personal responsibility for achievement to be able to feel pride. At the same time, appraising the situation as being at least partially responsible for the achievement increases feelings of self-inflation and perceived status. Moreover, when one other individual or two people are together responsible for the achievement, the pride experience generates stronger feelings of closeness compared to a group of people being responsible for the achievement. These findings suggest that both experienced responsibilities for the achievement and the number of people contributing to the achievement are essential to understand the subjective experience of pride. At the same time, future research is needed to further conceptualize and examine how responsibility and the number of achievers contribute to the distinction between group-pride and vicarious-pride. For example, whereas the manipulations of group-pride and vicarious-pride in our research were based on the definitions of these concepts in existing research, a critical look at pride research shows that scholars differ in their view on whether achievement of a group of other people concerns group-pride or vicarious-pride. A more well-developed conceptualization of group-pride and vicarious-pride is thus necessary to advance pride research.

Interestingly, our findings suggest that all forms of pride can motivate people toward future actions. Pride has been previously understood as one of the positive emotions that may motivate various kinds of positive behaviors (McCullough et al., 2001; Tangney et al., 2007), including perseverance (Williams and DeSteno, 2008; Wilcox et al., 2011), and prosocial behaviors (Haidt, 2003; Williams and DeSteno, 2009; Dorfman et al., 2014). At the same time, researchers are hesitant to present self-pride as a way to motivate positive behaviors because of its negative connotations. The present findings reveal that group-pride and vicarious-pride can also act as motivators, without the negative connotations of self-pride. We consider it therefore relevant for future research to investigate the influences of group-pride and vicarious-pride on different types of behavior, and to examine whether these emotions can be seen as fruitful motivators for positive behaviors in society.

### Limitations and Future Research

Parental pride may include a unique form of indirect responsibility that has not been captured in our studies (Nakamura, 2013; Pasupathi et al., 2020). Whereas our studies focused on either no, shared, or full direct responsibility for the positive outcome, parental pride shows that it is also possible to experience indirect responsibility for positive outcomes. For example, when a child performs well, parents may assign the achievements of their child to their upbringing or to the resources that they have provided. Consequently, parents may feel responsible, albeit indirectly, for the positive outcomes and therefore feel proud of themselves. Other examples may be coaches who feel proud of their sports team, or sponsors of election candidates winning the elections. Future research is poised to examine whether this indirect responsibility may be yet another aspect of responsibility that can contribute to feelings of pride, and if so, whether the experiential contents of this indirect form of pride resembles self-pride, group-pride, or vicarious-pride.

In our studies, we did not focus on the experience of hubristic pride (Tracy and Robins, 2007b) for multiple reasons. First, hubris or hubristic pride may concern a more individual tendency (Lewis, 1992) or a tendency to attribute achievements to personal abilities (Lange and Crusius, 2015; for an exception see Sullivan, 2013). In our research, we were instead interested in more situational experiences of pride, or attributions of the achievement to unstable causes. Second, although theoretically, it might be possible to experience pride over the achievements of others that are attributed to traits rather than efforts or luck, it is currently unclear how this experience would work. For example, when vicarious-pride is experienced, would it matter whether the achievement would be ascribed to the person having performed well or to the person containing a prestigious characteristic? Future research is needed to further examine this.

Group-pride or vicarious-pride have also been studied in a related concept called “basking-in-reflected-glory” (Cialdini et al., 1976; Harth et al., 2008). When people engage in basking-in-reflected-glory, they associate themselves with successful others, for example by wearing related apparel (Cialdini et al., 1976), using public displays of team support (Carter and Sanna, 2006; Miller, 2009), or using the plural “we” (Cialdini et al., 1976; Bernache-Assollant et al., 2007). Only a few studies have linked the findings of basking-in-reflected-glory to the findings of group-pride or vicarious-pride, or clarified how these concepts relate to each other (Harth et al., 2008, 2013). Our findings on the experiential contents of group-pride and vicarious-pride, as well as on the role of responsibility for the achievement and the number of achievers in the experience of pride, may provide some novel insights that can aid future research in distinguishing these related concepts.

With the current findings presenting the first path toward a distinction between the subjective experiences of the different forms of pride, future research may delve into potential moderators that may play a role in this process. It is not unlikely that the relationships found in the present research may depend on individual aspects, such as the need for achievement (McClelland and Liberman, 1949) or need for affiliation (Shipley and Veroff, 1952), or on cultural aspects, such as individualism or collectivism (Hofstede, 1980). Future research may disentangle how much the differences between the three forms of pride depend on individual and cultural factors.

Across our experiments, we have observed differences in the experiential contents of the three different forms of pride. One may wonder whether this signifies the existence of distinct emotions. The current state of the field of emotion research does not provide agreement on what constitutes distinct emotions. Therefore, we are hesitant to argue that self-pride, group-pride, and vicarious-pride are indeed divergent emotions. Also, the experiential contents of emotions are just one dimension of emotion, next to elements, such as cognitions, expressions, body sensations, and action readiness (Frijda, 1986, 2006). Claiming distinctiveness for self-pride, group-pride, and vicarious-pride would thus also require evidence for distinctiveness on these other dimensionsof pride.

Before closing, we would like to make two observations regarding our studies. The three experiments included different measures and different samples, thereby adding to the reliability and validity of our findings. At the same time, all of our studies included autobiographical recall procedures and self-report measures, which have their weaknesses. Autobiographical recall procedures rely on the memories of emotional events of people, which can be prone to goals and beliefs of people about themselves (Walker et al., 2003; Conway et al., 2004). At the same time, memories of pride experiences have been shown to be more detailed compared to memories of for example shame experiences (D’Argembeau and Van der Linden, 2008). Also, even though our studies used both student and non-student samples, all three samples consisted of mostly women. Some studies have found women to report less pride in achievements in specific domains than men (e.g., Magee, 2015), but a meta-analysis on gender differences in pride experiences across domains did not reveal any difference between women and men on the experiences of either authentic pride or hubristic pride (Else-Quest et al., 2012). Therefore, it currently seems unlikely that our findings would depend on the gender balance of our samples. Replications of our findings with different emotion inductions, more varied samples, and behavioral measures are necessary to advance our knowledge of self-pride, group-pride, and vicarious-pride.

## Conclusion

Previously, we knew a lot about the experience of pride for achievements that we are personally responsible for, but we knew very little about the experience of pride for shared achievements or for achievements that we are not responsible for. We now know that, although we can feel proud of any of those achievements, whether to have personally achieved something, to have together achieved something, or to have another person achieve something, *feels* different. These different feelings may have different consequences for oneself and others, thereby possibly changing our perspective of pride as a sinful emotion. Feeling proud together, or feeling proud of others, may thus be an experience worth fighting for.

## Data Availability Statement

The datasets presented in this study can be found in online repositories. The names of the repository/repositories and accession number(s) can be found below: aspredicted.org (link for every experiment provided in the manuscript).

## Ethics Statement

The studies involving human participants were reviewed and approved by the Tilburg University. The patients/participants provided their written informed consent to participate in this study.

## Author Contributions

Both authors listed have made a substantial, direct and intellectual contribution to the work, and approved it for publication.

## Conflict of Interest

The authors declare that the research was conducted in the absence of any commercial or financial relationships that could be construed as a potential conflict of interest.

## Publisher’s Note

All claims expressed in this article are solely those of the authors and do not necessarily represent those of their affiliated organizations, or those of the publisher, the editors and the reviewers. Any product that may be evaluated in this article, or claim that may be made by its manufacturer, is not guaranteed or endorsed by the publisher.

## Footnotes

1 In all analyses in Experiment 1 we checked whether being a Dutch or International student affected the outcomes. We observed two main effects of cultural background: Dutch students reported lower levels of authentic pride and of perceived status than International students. We did not observe any significant interaction between background and experimental condition.

2 The experiment formed part of the thesis project of two students, who conducted the research under supervision of the authors. The thesis students had also created items relating to social identity/closeness and negative affect. It is unclear which existing constructs/scales inspired their creation. In addition, these scales contained issues with face validity and reliability. Therefore, we decided not to include these scales in the current manuscript.

3 This study took place in the first 3 weeks of April 2020, immediately after the pandemic hit the country. Therefore, our instruction in the Control condition referred specifically to a regular weekday before COVID-19 and indicated that the friend or family member should still be alive today (as we did not wish to induce any negative emotions).

4 Based on previous research (e.g., Van Osch et al., 2019) we employed both a single-item measure of pride, and the presented three-item scale to assess pride. In all experiments, both measures revealed identical results. Therefore, in the results sections we present the results of the single-item measure for pride.

5
https://aspredicted.org/blind.php?x=tb8pe9

6 The words “benijden” and “afgunst” were used in Dutch, which specifically refer to experiences of benign and malicious envy (see Van de Ven, 2017). After data collection we realized that the single item for *admiration* (based on Van de Ven, 2017) was also part of the *other-appreciation* scale we had created for this experiment. We analyzed the scale and item as we had originally intended to (the same holds for Experiment 3).

7
https://aspredicted.org/blind.php?x=zd4gh7
